# The natural history, clinical outcomes, and genotype–phenotype relationship of otoferlin-related hearing loss: a systematic, quantitative literature review

**DOI:** 10.1007/s00439-023-02595-5

**Published:** 2023-09-07

**Authors:** Charles L. Ford, William J. Riggs, Tera Quigley, Orion P. Keifer, Jonathon P. Whitton, Vassili Valayannopoulos

**Affiliations:** Decibel Therapeutics, Inc, Boston, MA USA

## Abstract

Congenital hearing loss affects one in 500 newborns. Sequence variations in *OTOF*, which encodes the calcium-binding protein otoferlin, are responsible for 1–8% of congenital, nonsyndromic hearing loss and are the leading cause of auditory neuropathy spectrum disorders. The natural history of otoferlin-related hearing loss, the relationship between *OTOF* genotype and hearing loss phenotype, and the outcomes of clinical practices in patients with this genetic disorder are incompletely understood because most analyses have reported on small numbers of cases with homogeneous *OTOF* genotypes. Here, we present the first systematic, quantitative literature review of otoferlin-related hearing loss, which analyzes patient-specific data from 422 individuals across 61 publications. While most patients display a typical phenotype of severe-to-profound hearing loss with prelingual onset, 10–15% of patients display atypical phenotypes, including mild-to-moderate, progressive, and temperature-sensitive hearing loss. Patients’ phenotypic presentations appear to depend on their specific genotypes. For example, non-truncating variants located in and immediately downstream of the C_2_E calcium-binding domain are more likely to produce atypical phenotypes. Additionally, the prevalence of certain sequence variants and their associated phenotypes varies between populations due to evolutionary founder effects. Our analyses also suggest otoacoustic emissions are less common in older patients and those with two truncating *OTOF* variants. Critically, our review has implications for the application and limitations of clinical practices, including newborn hearing screenings, hearing aid trials, cochlear implants, and upcoming gene therapy clinical trials. We conclude by discussing the limitations of available research and recommendations for future studies on this genetic cause of hearing loss.

## Introduction

Hearing loss is the fourth leading cause of disability worldwide, affecting an estimated 6.8% of the global population (Neumann et al. 2019). One out of every 500 children is born with hearing loss, and approximately half of these cases have severe-to-profound bilateral hearing loss (Lieu et al. 2020). The majority of congenital hearing loss cases are caused by genetic variations (Shearer et al. 1999; Lieu et al. 2020). Sequence variations in the *OTOF* gene, which encodes the membrane-associated protein otoferlin, are responsible for 1–8% of congenital, nonsyndromic hearing loss cases and are the leading cause of auditory neuropathy spectrum disorders (ANSD; Azaiez et al. 1993). Otoferlin is a calcium-sensing protein involved in inner hair cell (IHC) vesicular transport and exocytosis, which are critical processes for IHC signaling to auditory nerve fibers (Michalski et al. 2017). IHCs and spiral ganglion neurons form specialized ribbon synapses for transmitting acoustic signals with high temporal fidelity. This fidelity is critical for certain aspects of auditory perception, including sound localization and speech comprehension (Nouvian et al. 2006). *OTOF* sequence variants can result in deficient or non-functional otoferlin protein, thus disrupting synaptic transmission and causing otoferlin-related hearing loss.

Also known as DFNB9, otoferlin-related hearing loss follows an autosomal recessive inheritance pattern and typically presents as severe-to-profound sensorineural hearing loss (SNHL) with congenital or prelingual onset (Azaiez et al. 1993). However, the literature is replete with reports of atypical hearing phenotypes, including mild-to-moderate (Yildirim-Baylan et al. 2014; Fedick et al. 2016; Wang et al. 2018; Santarelli et al. 2021; Iwasa et al. 2022), progressive (Chiu et al. 2010; Matsunaga et al. 2012; Fedick et al. 2016), and even temperature-sensitive (Varga et al. 2006; Marlin et al. 2010; Zhang et al. 2016b; Zhu et al. 2021) hearing loss. The latter occurs when an individual’s auditory threshold varies with fluctuations in body temperature. Importantly, the tone-detection thresholds used to determine the severity of hearing loss do not always correlate with functional deficits. As in other forms of ANSD, speech comprehension in otoferlin-related SNHL can be poorer than expected, based on hearing thresholds in less severe phenotypes (Santarelli et al. 2013; Vona et al. 2020).

The phenotypic spectrum of otoferlin-related SNHL is likely a consequence of the diversity of *OTOF* sequence variants. Over 200 pathogenic and likely pathogenic *OTOF* variants have been reported, yet the relationship between phenotype and *OTOF* genotype is poorly understood (Vona et al. 2020). Consisting of 48 exons spanning 90 kb of DNA, *OTOF* yields five isoforms in humans (Yasunaga et al. 2000). The canonical otoferlin isoform in human cochlea contains six C_2_ domains, which likely bind calcium or interact with proteins, one putative C_2_ domain, at least one Fer domain hypothesized to interact with phospholipid membranes, and a transmembrane domain (Yasunaga et al. 2000; Pangršič et al. 2012; Harsini et al. 2018; Vona et al. 2020; Dominguez et al. 2022; Liu et al. 2023). These domains subserve otoferlin’s functions in vesicular tethering, exocytosis, and replenishment at the presynaptic axon terminals of IHCs (Johnson and Chapman 2010; Helfmann et al. 2011; Padmanarayana et al. 2014; Meese et al. 2017; Michalski et al. 2017; Vona et al. 2020; Liu et al. 2023). However, the precise structure of otoferlin is still being elucidated—recent studies identified a previously unannotated exon (Ranum et al. 2019) and a novel truncated isoform in mouse IHCs (Liu et al. 2023), and a recent in silico analysis suggested the existence of a novel “C2-FerA” domain (Dominguez et al. 2022).

Although *OTOF* variants can disrupt the physiology of synaptic transmission at the ribbon synapse (Roux et al. 2006; Santarelli et al. 2009, 2011, 2015; Santarelli 2010), their impact on cochlear anatomy may be minimal. There is evidence that the IHCs, outer hair cells (OHCs), and auditory nerve develop normally, at least initially. In mouse models of otoferlin deficiency, early postnatal development of IHCs and ribbon synapses appears normal, although the quantity and morphology of ribbon synapses are later altered (Roux et al. 2006; Al-Moyed et al. 2019; Stalmann et al. 2021). Many patients with otoferlin-related SNHL initially have preserved otoacoustic emissions (OAEs), which indicate that OHCs are present and functional (Varga et al. 2003; Iwasa et al. 2022). However, multiple reports indicate that OAEs appear to diminish in amplitude and eventually become absent over time, indicating that OHCs may gradually die or become non-functional as a result of *OTOF* sequence variants (Rodríguez-Ballesteros et al. 2003; Loundon et al. 2005; Rouillon et al. 2006; Chiu et al. 2010; Kitao et al. 2019; Thorpe et al. 2022).

Due to the initial preservation of OAEs, children with otoferlin-related hearing loss commonly pass newborn hearing screening (NBHS) based only on OAE detection (Varga et al. 2003; Iwasa et al. 2022). Upon identification of SNHL, hearing aids are recommended as first-line intervention (Azaiez et al. 1993). However, with the sound-amplifying OHCs initially intact, additional sound amplification from hearing aids may not compensate for dysregulated exocytosis downstream at the ribbon synapse (Rouillon et al. 2006; Chiu et al. 2010; Marlin et al. 2010; Santarelli et al. 2021). Hearing aids have also been hypothesized to accelerate the death of OHCs and the loss of OAEs in these patients through acoustic trauma mechanisms (Rouillon et al. 2006; Kitao et al. 2019; Vona et al. 2020). In contrast, cochlear implants are expected to provide clinical benefit in most patients with otoferlin-related SNHL because they bypass the dysfunctional ribbon synapse to stimulate the auditory nerve directly (Rouillon et al. 2006; Zheng and Liu 2020; Iwasa et al. 2022). Conversely, patients with an ANSD phenotype [preserved OAEs and abnormal auditory brainstem response (ABR)] arising from postsynaptic dysfunction are less likely to benefit from cochlear implantation (Shearer et al. 2017; De Siati et al. 2020). For this reason, in the absence of an identified genetic etiology that indicates presynaptic dysfunction, cochlear implantation may be delayed in patients with ANSD. In the future, genetic therapies that restore functional otoferlin to IHCs may offer patients with *OTOF*-related SNHL more natural hearing than cochlear implants can provide (Akil et al. 2019; Al-Moyed et al. 2019; Vona et al. 2020). Regardless of the clinical interventions under consideration, optimal patient care and translational research require thoroughly understanding the relationship between *OTOF* variants, phenotypic manifestations, and clinical outcomes.

Despite numerous case reports and small-scale studies of *OTOF*-related hearing loss, large-scale studies are scarce, and there is an absence of comprehensive meta-analyses or quantitative literature reviews. Vona et al. (2020) provide an excellent qualitative description of the genetic and phenotypic heterogeneity of otoferlin-related SNHL, and Thorpe et al. (2022) analyzed some aspects of the genotype–phenotype relationship by combining original data with data from the literature. However, the natural history, genotype–phenotype relationships, and clinical prognoses of this disorder remain incompletely defined due to a lack of quantitative analyses in large cohorts containing the full genetic spectrum of *OTOF*-related SNHL. Here, we attempt to elucidate these aspects of otoferlin-related SNHL by conducting a systematic, quantitative literature review. The resulting analyses provide a thorough representation of phenotypes, associated genotypes, and clinical outcomes reported in the literature.

## Methods

### Search strategy

Original article search was completed using the search term “OTOF” in the PubMed online database (https://pubmed.ncbi.nlm.nih.gov) on February 14, 2022. An abstract scan requiring human data yielded 109 unique publications. After the original query date, two additional papers (Iwasa et al. 2022; Lee et al. 2022) were published and identified via a Pubmed database alert for new articles, yielding a total of 111 publications.

### Case and publication selection

**Fig. 1:**
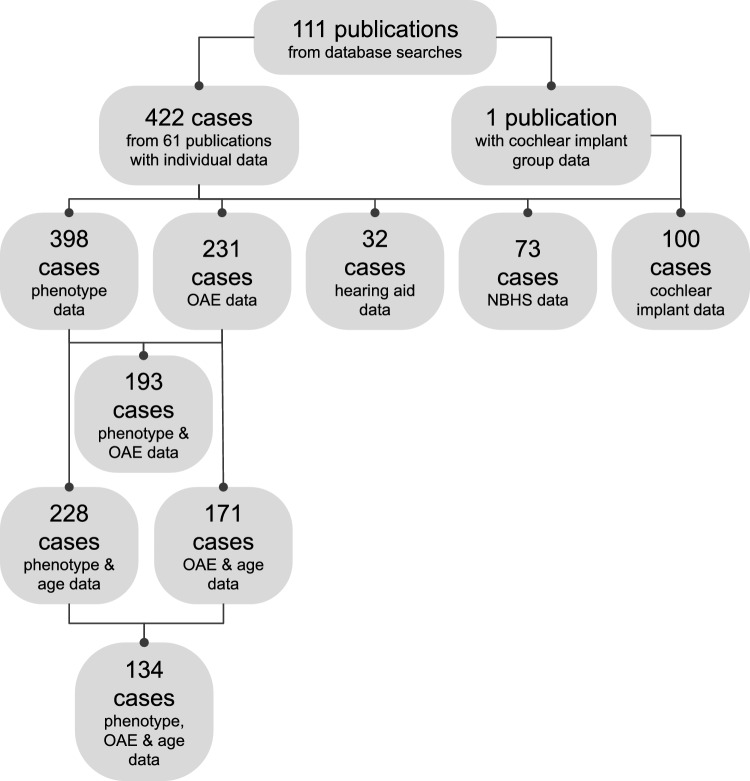
Data collection flow diagram. From the 111 publications identified in our initial database search, we analyzed patient-specific data on 422 cases with biallelic *OTOF* variants across 61 publications, in addition to group data from one publication. *OAE* otoacoustic emissions, *NBHS* newborn hearing screening

The 111 identified publications were reviewed for data on individuals with otoferlin-related hearing loss. Inclusion criteria included: non-syndromic SNHL; biallelic, pathogenic or likely pathogenic *OTOF* sequence variations confirmed via sequencing; individual data on genotype, phenotype, OAEs, or clinical outcomes. Exclusion criteria included: syndromic or conductive hearing loss; severe prematurity or perinatal hypoxia; treatment with ototoxic agents; perinatal jaundice or hyperbilirubinemia; traumatic brain injury; noise exposure; anatomical abnormalities of the auditory or vestibular systems. One patient with cystic fibrosis, one with osteogenesis imperfecta, and one with type I diabetes were included as these conditions were not expected to have influenced their hearing phenotypes. Cases duplicated between publications were combined into a single entry, though due to unclear language in some publications, we cannot exclude the possibility that a small number of duplicated cases were included in our review. In total, 422 individual patients from 61 publications were included (Fig. 1; Table 1). One additional publication (Lee et al. 2022) with group data, but no individual data, showing speech perception outcomes after cochlear implantation was included in our cochlear implant analysis. All analyses were performed and visualized using GraphPad Prism (version 9.5.0, GraphPad Software, San Diego, CA, USA).

**Table 1:** Included publications

Publication	Cases	Country of origin
Ahmed et al. (2021)	7	Pakistan
Alkowari et al. (2017)	2	Qatar
Almontashiri et al. (2018)	7	Saudi Arabia
Ammar-Khodja et al. (2015)	7	Algeria
Bai et al. (2019)	1	China
Bitarafan et al. (2020)	1	Iran
Safka Brozkova et al. (2020)	2	Czech Republic
Chiu et al. (2010)	6	Taiwan
Choi et al. (2009)	55	Pakistan
Churbanov et al. (2016)	2	Russia
Dallol et al. (2016)	2	Saudi Arabia
de Oliveira Costa et al. (2012)	2	Brazil
Fareed et al. (2021)	2	India
Fedick et al. (2016)	4	USA
Gallo-Terán et al. (2004)	4	Spain
Gallo-Terán et al. (2006)	1	Spain
Houseman et al. (2001)	5	United Arab Emirates
Hutchin et al. (2005)	2	United Kingdom
Iwasa et al. (2013)	7	Japan
Iwasa et al. (2019)	39	Japan
Iwasa et al. (2022)	64	Japan
Jin et al. (2014)	3	Korea
Kim et al. (2018)	13	Korea
Lee et al. (2020)	2	Korea
Li et al. (2021)	4	China
Likar et al. (2018)	1	Slovenia
Loundon et al. (2005)	1	France
Marlin et al. (2010)	3	France
Matsunaga et al. (2012)	13	Japan
Naz et al. (2017)	7	Pakistan
Noman et al. (2019)	4	Pakistan
Pandey et al. (2017)	1	India
Qiu et al. (2019)	2	China
Rodríguez-Ballesteros et al. (2003)	21	Spain
Rodríguez-Ballesteros et al. (2008)	36	Argentina, Colombia, Spain
Romanos et al. (2009)	3	Brazil
Rouillon et al. (2006)	2	France
Runge et al. (2013)	2	USA
Santarelli et al. (2009)	1	Italy, Spain
Santarelli et al. (2011)	1	Italy, Spain
Santarelli et al. (2015)	7	Italy, Spain
Santarelli et al. (2021)	5	Italy, Spain
Shahin et al. (2010)	3	Pakistan
Souissi et al. (2021)	1	Tunisia
Tabatabaiefar et al. (2018)	1	Iran
Tang et al. (2017)	2	China
Tekin et al. (2005)	3	Turkey
Varga et al. (2003)	3	USA
Varga et al. (2006)	4	USA
Wang et al. (2010)	1	China
Wang et al. (2018)	4	China
Wu et al. (2018)	10	China
Wu et al. (2020)	1	China
Xia et al. (2018)	2	China
Xiang et al. (2020)	1	China
Yildirim-Baylan et al. (2014)	9	Turkey
Zadro et al. (2010)	4	Italy
Zhang et al. (2013)	1	China
Zhang et al. (2016a)	3	China
Zhang et al. (2016b)	11	China
Zhu et al. (2021)	4	China

All 61 publications contained patient-specific data on individuals with confirmed pathogenic or likely-pathogenic, biallelic *OTOF* sequence variations. In total, data were collected on 422 unique individuals from these publications

### Phenotype analyses

To delineate patient phenotypes, we either (1) calculated the air-conduction, pure-tone average hearing thresholds determined by behavioral or electrophysiological audiometry at 0.5, 1, 2, and 4 kHz between ears, or (2) used the average hearing threshold reported by the authors. When a range of hearing thresholds was reported, we used the mean of that range. If a range was unbounded at the upper end (e.g., ≥ 80 dB), we used the lower limit (e.g., 80 dB). We then assigned a hearing phenotype to each individual using a simplified version of the classifications established by Clark (1981): 0–25 dB, normal; 26–40 dB, mild; 41–70 dB, moderate; 71–90 dB, severe; > 90 dB, profound. If a numeric hearing threshold was not reported, we accepted the severity classification assigned by the authors. Patients reported as “severe-profound” (n = 59) were classified as severe, those with thresholds below 25 dB but with complaints of hearing deficits were classified as mild (n = 5), and those with fluctuant hearing thresholds that were neither temperature-sensitive nor unidirectionally progressive were classified as unstable (n = 2). ABRs were classified as absent if no response was detected at the maximum stimulation level, and they were classified as abnormal if an aberrant response was detected in at least one ear. No ABRs were described as normal. The results of ABR-based NBHS were only included in NBHS analyses, not in other ABR analyses.

### Genotype analyses

Deletions larger than one residue, nonsense, frameshift, and splice-site variants were considered truncating (T) unless specifically described as non-truncating (n = 1). Single-residue, in-frame deletions and missense variants were classified as non-truncating (NT). In cases of multiple homozygous variants, the most severe variant was used to classify subjects. We defined the boundaries of functional regions within otoferlin according to Vona et al. (2020) and, for the putative C_2_ domain, C_2_de, according to Pangršič et al. (2012). One NT splice-site variant was not included in the regional analysis. Assessments of pathogenicity were multi-factorial and included other reports of the variant, sequence conservation, co-segregation of the variant with SNHL, results of mutation prediction software reported by authors, and exclusion of other possible genetic and environmental causes of hearing loss.

### Otoacoustic emissions analyses

Distortion product otoacoustic emissions (DPOAEs) and transient evoked otoacoustic emissions (TEOAEs) were all categorized as “OAEs.” OAE results were classified as “present,” “absent,” “reduced,” or, in cases where OAEs were lost over time, “present, then absent.” “Present” was used when (1) qualitatively reported by the authors or, if raw data were provided, (2) OAEs were present (greater than 5 dB signal-to-noise ratio) at 50% or more of the frequencies tested in both ears. “Reduced” was used when (1) both ears had present OAEs in less than 50% of frequencies or (2) only one ear had present OAEs in at least 50% of frequencies. “Absent” was used when (1) described qualitatively by the authors or (2) if raw data indicated absent (less than 5 dB signal-to-noise ratio) OAE responses at all tested frequencies. The results of OAE-based NBHS were only included in NBHS analyses, not in other OAE analyses.

### Analyses of clinical outcomes

We compared the results of NBHS that utilized ABRs, OAEs, and unspecified test methodology. Hearing aid outcomes were reported, predominantly in subjective terms, for 32 patients from 12 publications, which we summarized in Table 3. Methodological variability in cochlear implant outcome assessments limited our ability to conduct quantitative analyses, so we summarized the reported outcomes of 100 patients from 17 publications in Table 4.

### Analyses involving age

Age data were used only if a specific age was indicated for the case. Age ranges were not used unless the entire range fit within a single bin. If hearing thresholds were measured at multiple ages, we used the youngest age. If OAEs were measured and present at multiple ages, we used the oldest age.

## Results

### Phenotypes

**Fig. 2:**
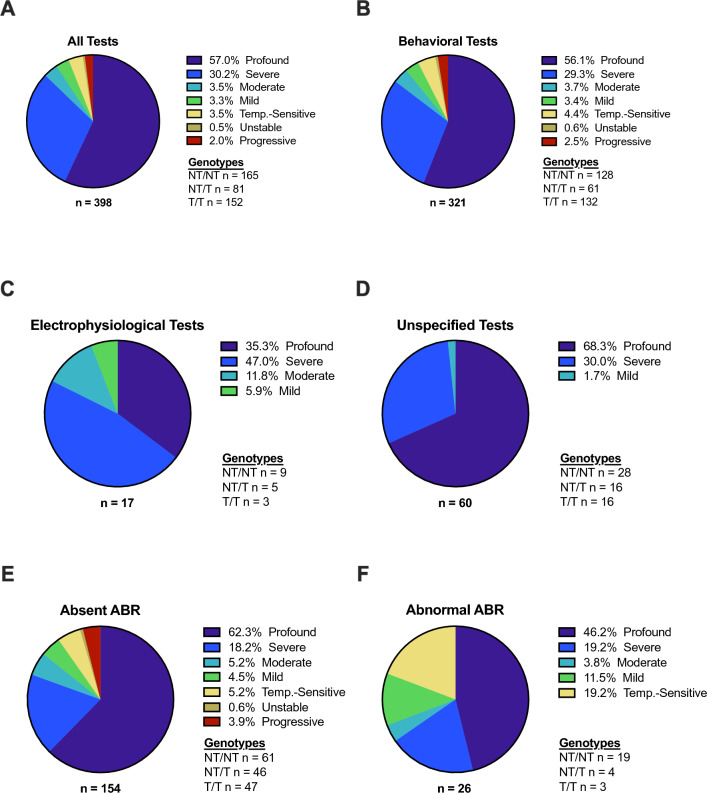
Phenotypes. The spectrum of reported phenotypes in otoferlin-related sensorineural hearing loss (**A**) was subdivided into phenotypes classified with behavioral audiometry (**B**), electrophysiological audiometry (**C**), and audiometry with unspecified methodology (**D**). The phenotypes of cases with absent auditory brainstem responses (ABRs) at the maximum stimulation tested are shown in (**E**) while the phenotypes of cases with abnormal ABRs are shown in (**F**). Phenotype classification was assigned based on hearing thresholds as follows: 0–25 dB, normal; 26–40 dB, mild; 41–70 dB, moderate; 71–90 dB, severe; > 90 dB, profound. *NT* non-truncating, *T* truncating

**Fig. 3:**
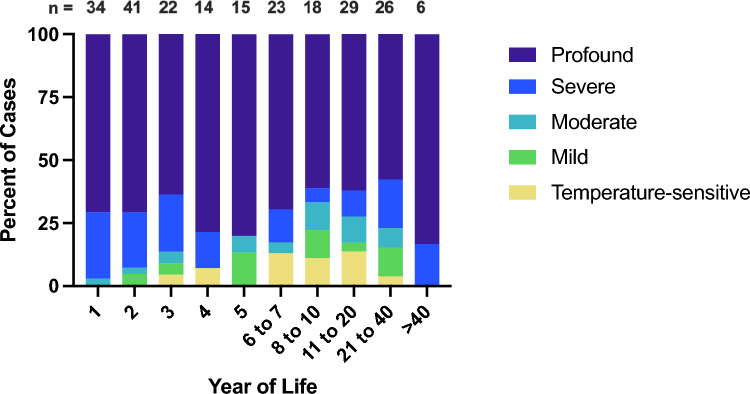
Phenotypes by age. The distribution of hearing phenotypes reported in patients of different ages is shown. The majority of cases in all age groups have severe-to-profound sensorineural hearing loss. The relative paucity of temperature-sensitive, mild, and moderate cases at younger ages is likely a consequence of delayed diagnoses rather than a physiological process. Unstable and progressive cases were omitted due to their variability over time. Year of Life 1 = birth to 1 year old; Year of Life 2 = 1.01 years old to 2 years old; etc.

Audiometry results were available for 398 of the 422 individuals with biallelic, pathogenic or likely pathogenic *OTOF* variants (Fig. 2A). Of those cases, 227 (57%) displayed profound SNHL and 120 (30%) displayed severe SNHL, which are both typical phenotypes. The remaining 51 (13%) patients displayed atypical phenotypes—SNHL was moderate in 14 (3.5%), mild in 13 (3.3%), temperature-sensitive in 14 (3.5%), unstable in two (0.5%), and progressive in eight (2%). These proportions changed minimally when only patients assessed with behavioral audiometry (e.g., pure tone audiometry, visual reinforcement audiometry) were analyzed (n = 321; Fig. 2B) and patients assessed with electrophysiological tests (e.g., ABR audiometry, auditory steady-state response audiometry; n = 17; Fig. 2C) and unspecified methodology (n = 60; Fig. 2D) were excluded. Atypical phenotypes were present in 30 of 154 (19%) cases with absent ABRs at the maximum stimulation tested (Fig. 2E) and in nine of 26 (35%) cases with abnormal ABRs (Fig. 2F). Severe or profound SNHL was present in 70–100% of cases in all age groups (Fig. 3).

### Genotypes

**Fig. 4:**
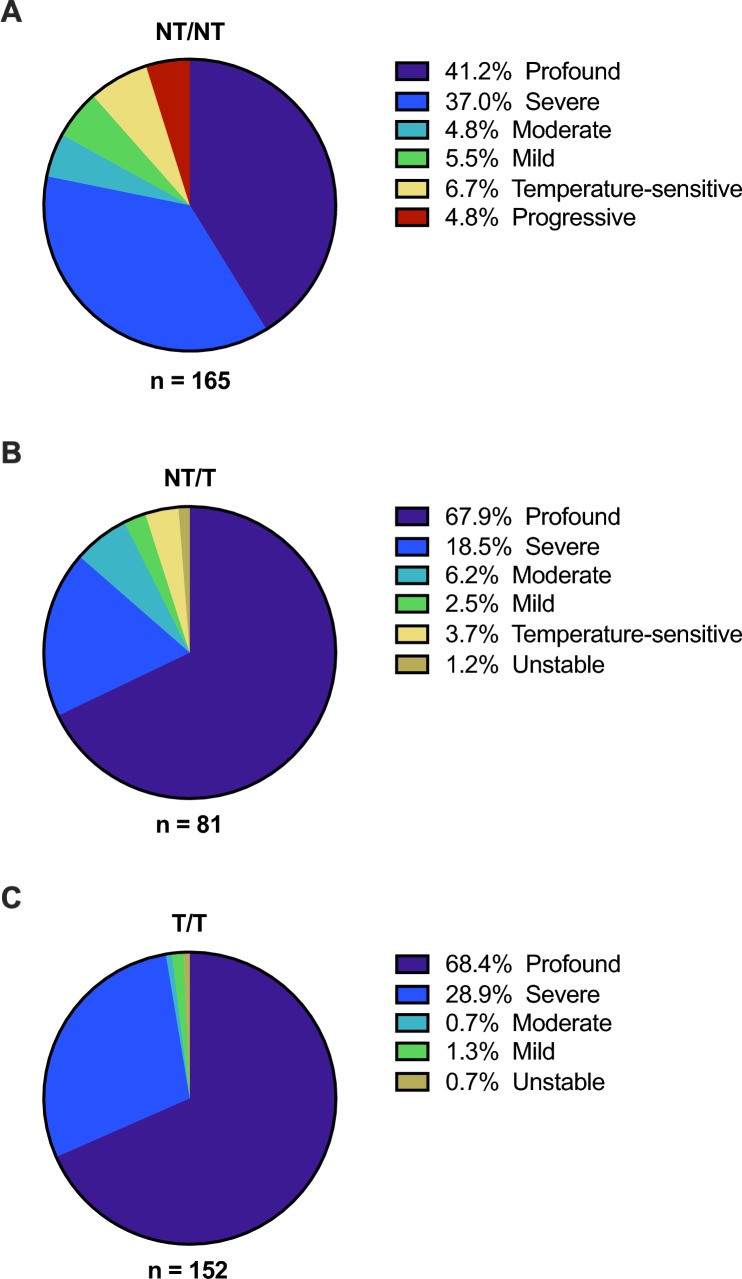
Phenotypes by genotype. The spectrum of phenotypes in cases with biallelic *OTOF* variants was subdivided according to genotype. Twenty-two percent of cases with two non-truncating alleles (NT/NT) had atypical (mild, moderate, temperature-sensitive, progressive, or unstable) phenotypes (**A**). Atypical phenotypes were present in 14% of cases with one non-truncating and one truncating allele (NT/T; **B**), and in 2.7% of cases with two truncating alleles (T/T; **C**). Hearing thresholds: 0–25 dB, normal; 26–40 dB, mild; 41–70 dB, moderate; 71–90 dB, severe; > 90 dB, profound

Of the 398 cases with biallelic, pathogenic or likely pathogenic *OTOF* variants and phenotypic data, 238 (60%) were homozygotes and 160 (40%) were compound heterozygotes. One hundred and sixty-five cases (41%) had two NT variants (NT/NT; Fig. 4A), 81 (20%) had one NT and one T variant (NT/T; Fig. 4B), and 152 (38%) had two T variants (T/T; Fig. 4C). Typical profound or severe phenotypes were reported in 129 (78%) of 165 NT/NT cases, while the remaining 36 (22%) displayed atypical phenotypes, including 11 (6.7%) with temperature-sensitive SNHL and 8 (4.8%) with progressive SNHL (Fig. 4A). In cases with NT/T genotypes (n = 81), 70 (86%) had profound or severe SNHL, while 11 (14%) displayed atypical phenotypes (Fig. 4B). Among T/T cases (n = 152), 148 (97%) displayed severe or profound SNHL, while only four (2.7%) had atypical phenotypes (Fig. 4C).

**Fig. 5:**
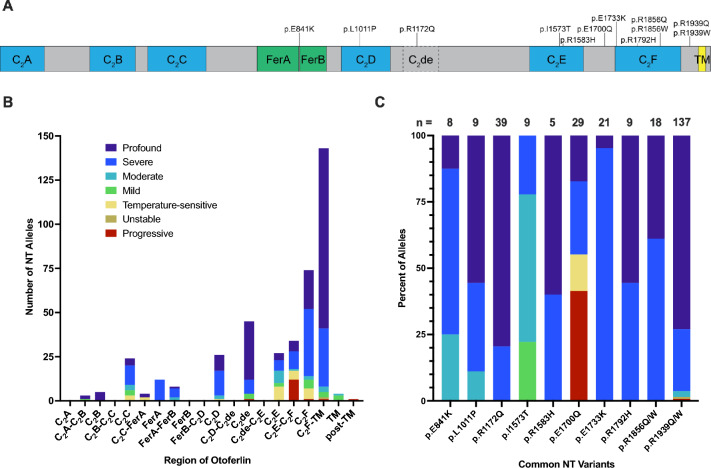
Phenotypes by non-truncating variant. **A** Shows a schematic of otoferlin with specific domains demarcated and the locations of common non-truncating (NT) alleles. **B** Shows each reported instance of a NT allele within each gene region, as well as the phenotype of the patient, such that the phenotypes of cases with two NT alleles are represented twice (once for each allele) and the phenotypes of cases with one NT allele and one truncating allele are represented once. Pathogenic NT variants were frequently reported in some regions (e.g., C_2_F, C_2_F-TM) and not reported in others (e.g., C_2_A, FerB), and NT variants were more commonly associated with atypical phenotypes in some regions (e.g., C_2_E) than in others (e.g., C_2_F-TM). **C** Shows the most common NT alleles in our dataset and their associated phenotypes (each allele is represented once, so the phenotypes of homozygous cases are represented twice). Variant p.I1573T is notable for its frequent association with mild-to-moderate sensorineural hearing loss, whereas p.E1700Q is commonly associated with progressive or temperature-sensitive sensorineural hearing loss. C_*2*_*A, C*_*2*_*B, C*_*2*_*C, C*_*2*_*D, C*_*2*_*E, and C*_*2*_*F* C_2_ domains, *C*_*2*_*de* putative C_2_ domain, *FerA* *FerB* ferlin domains, *TM* transmembrane domain

We next investigated whether certain NT variants, or NT variants in certain regions of the gene, might be associated with particular phenotypes (Fig. 5). A schematic of otoferlin protein is shown in Fig. 5A with the locations of functional domains demarcated and common NT variants indicated. Figure 5B shows the number of times NT alleles were reported within each gene region, and the phenotype associated with each allele (e.g., the phenotype of each NT/NT case is represented twice, once for each allele, while NT/T cases are represented once). The majority of NT alleles (total n = 410) were localized to either a C_2_ domain (n = 201; 49%) or the regions flanking the final C_2_ domain, C_2_F (n = 177; 43%). Twenty percent (n = 83) of all NT alleles (n = 410) were associated with atypical phenotypes, but in the C_2_E domain and the region immediately downstream (C_2_E-C_2_F), 57% (n = 35 of 61) of NT alleles were associated with atypical phenotypes. Figure 5C shows the most common NT alleles in our dataset, which were reported in at least five individuals and two unrelated families, and the frequency of phenotypes associated with each allele (each allele is represented once, so the phenotypes of homozygous cases are represented twice). Notably, 78% of p.I1573T alleles (n = 9) were associated with mild or moderate phenotypes, and 41% and 14% of p.E1700Q alleles (n = 29) were associated with progressive or temperature-sensitive phenotypes, respectively. Ten variants in total were associated with temperature-sensitive phenotypes: p.G541S (Matsunaga et al. 2012; Zhang et al. 2016b), p.G614E (Romanos et al. 2009), p.R698T (Zhang et al. 2016a), p.R1080P (Romanos et al. 2009), p.R1157Q (Marlin et al. 2010), p.R1607W (Wang et al. 2010; Zhang et al. 2016b), p.P1628T (Zhu et al. 2021), p.E1661K (Zhang et al. 2016a), p.E1700Q (Zhu et al. 2021), and p.E1804del (Marlin et al. 2010). With the exception of p.E1700Q, these variants were exclusively identified in temperature-sensitive cases within our dataset.

As common allelic variants are not evenly distributed across different populations and geographic regions, we tabulated the number and locations of NT and T alleles reported in at least five individuals and two families from our dataset (Table 2).

**Table 2:** Geographic distribution of common variants

Variant	Alleles	Country
Non-truncating		
p.E841K	8	Korea, China
p.L1011P	9	Turkey, Korea
p.R1172Q	50	Japan
p.I1573T	9	Turkey, Japan
p.R1583H	7	China, Japan
p.E1700Q	29	China
p.E1733K	21	Pakistan, China
p.R1792H/C	11	Saudi Arabia, Japan
p.R1856Q/W	18	Pakistan, Japan, Czech Republic, Korea
p.R1939Q/W	142	Japan, Korea, Pakistan
Truncating		
p.R237X	16	United Arab Emirates, Algeria
p.R425X	13	Pakistan, China
p.Y474X	16	Japan
p.R708X	31	Pakistan, Algeria, India, Spain, Colombia
p.E747X	12	Qatar, Saudi Arabia, Libya, Italy
p.Q829X	94	Spain, Colombia, Argentina, France, USA
p.Y1064X	7	Japan, Korea, Czech Republic
p.Q1072X	5	Japan
c.3289-1G>T	20	Pakistan

The geographic locations of the most common sequence variations in our dataset are listed. All variants were reported in at least five individuals and two unrelated families

### Otoacoustic emissions (OAEs)

**Fig. 6:**
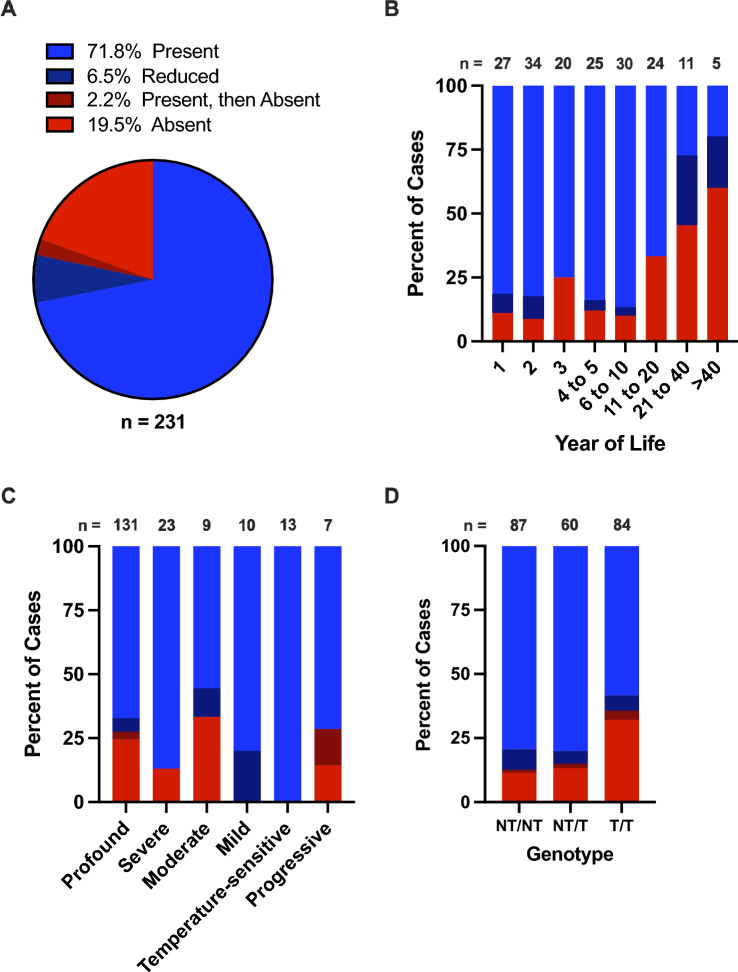
Otoacoustic emissions. Otoacoustic emissions (OAEs) were present, present but reduced, or initially present and lost over time in over 80% of cases (**A**). The proportion of cases with present OAEs decreases in older age groups (**B**; n = 5 cases with OAEs that were initially present and lost over time are represented twice, once at their oldest age with present OAEs and once at their youngest age with absent OAEs). Patients with mild or temperature-sensitive sensorineural hearing loss were more likely to have present OAEs than patients with other phenotypes (**C**), and patients with at least one non-truncating (NT) allele were more likely to have present OAEs than patients with two truncating (T) alleles (**D**). Only hearing loss phenotypes classified using behavioral audiometry were included in **C**. Year of Life 1 = birth to 1 year old; Year of Life 2 = 1.01 years old to 2 years old; etc.

**Fig. 7:**
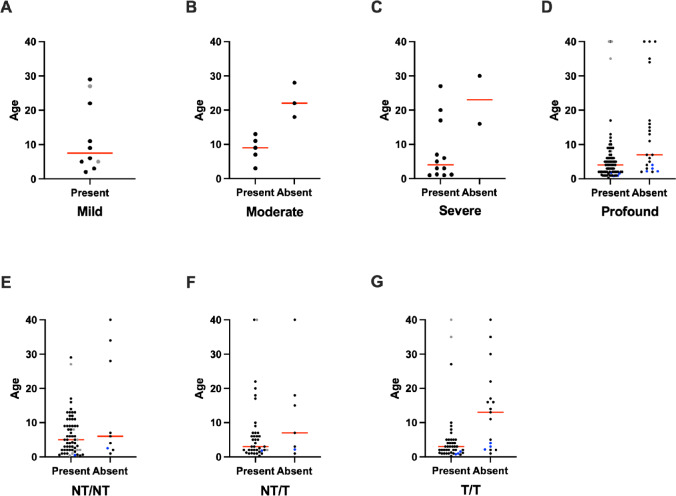
Otoacoustic emissions by age and phenotype or genotype. The age in years and results of otoacoustic emission (OAE) testing are shown for individuals with mild (**A**), moderate (**B**), severe (**C**), and profound (**D**) sensorineural hearing loss, and for individuals with two non-truncating (NT) alleles (**E**), one NT and one truncating (T) allele (**F**), and two T alleles (**G**). Individuals with reduced OAEs are represented as gray dots in the “Present” column, and individuals with present OAEs at one time and absent OAEs at a later time are represented twice, once in the “Present” and once in the “Absent” column, with blue dots. Red lines indicate the median age. Only hearing loss phenotypes classified using behavioral audiometry were included in **A**–**D**

Of the 231 patients with OAE data, OAEs were present in 166 (72%) and absent in 45 (19%; Fig. 6A). In 15 (6.5%) patients, OAEs were present but significantly reduced or only detected unilaterally. In five (2.2%) patients, OAEs were present at one time and later found to be absent; these patients were reported twice in analyses involving age—once at the oldest age with present OAEs and once at the youngest age with absent OAEs (Fig. 6B, n = 5; Fig. 7D, n = 4; Fig. 7E, n = 1; Fig. 7F, n = 1; Fig. 7G, n = 3). It should be noted, however, that the majority of publications only tested OAEs at a single time point and classified them as either “present” or “absent” without a “reduced” classification. Only patients assessed with behavioral audiometry were included in analyses involving OAEs and phenotype (Figs. 6C, 7A–D). While OAEs were absent in less than 25% of cases up to 10 years of age, OAEs were absent in 33% of patients aged 11–20, absent in 45% and reduced in 27% of patients aged 21–40, and absent in 60% and reduced in 20% of patients aged more than 40 years (Fig. 6B). Regarding phenotypes, OAEs were detected in all mild and temperature-sensitive cases and in approximately 75% of profound, severe, moderate, and progressive cases (Fig. 6C). Similarly, OAEs were present in approximately 80% of NT/NT and NT/T cases, but only in 58% of T/T cases (Fig. 6D).

Next, we examined the distribution of present vs. absent OAEs across ages in patients with specific phenotypes (Fig. 7A–D) and genotypes (Fig. 7E–G). To facilitate data visualization, cases with “reduced” OAEs are shown as gray dots within the “present” column, cases represented twice—once with present and once with absent OAEs—are shown as blue dots, and ages greater than  40 years are presented as 40. Sample sizes were limited for patients with OAE data, age data, and either mild (n = 10 present, n = 0 absent; Fig. 7A), moderate (n = 5 present, n = 3 absent; Fig. 7B), or severe phenotypes (n = 12 present, n = 2 absent; Fig. 7C), or NT/NT (n = 61 present, n = 9 absent) or NT/T (n = 39 present, n = 7 absent) genotypes. However, patients with profound SNHL and present OAEs (n = 79) tended to be younger (median age four years) than patients with profound SNHL and absent OAEs (n = 23; median age seven years; Fig. 7D), and patients with T/T genotypes and present OAEs (n = 43) tended to be younger (median age three years) than those with absent OAEs (n = 17; median age 13 years; Fig. 7G).

### Clinical outcomes

**Fig. 8:**
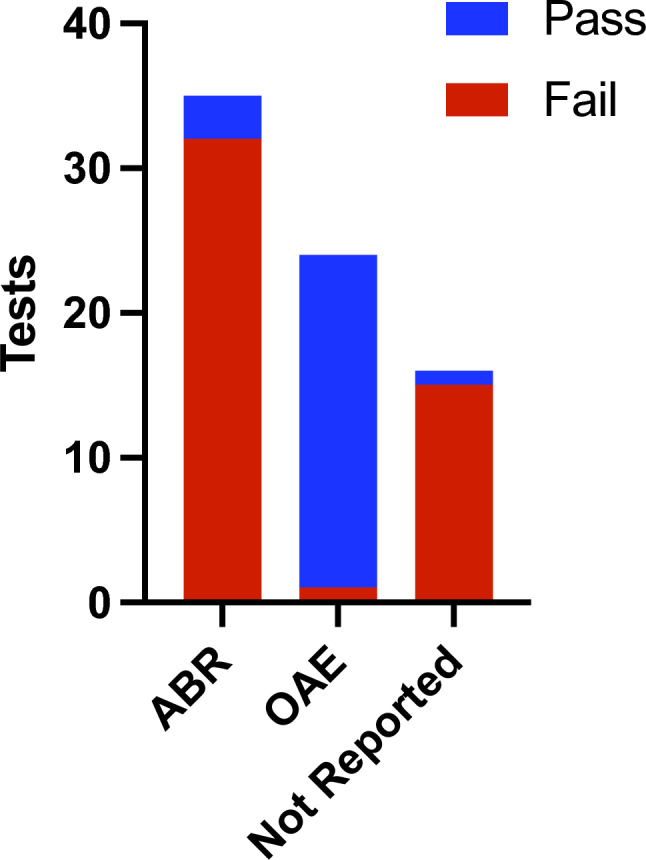
Newborn hearing screening outcomes. The results of auditory brainstem response (ABR)-based and otoacoustic emission (OAE)-based newborn hearing screenings (NBHS), as well as those with unspecified methodology, are shown. Twenty-three of 24 (96%) OAE-based NBHS were inappropriately passed compared to three of 35 (8.6%) ABR-based tests. Two patients underwent both OAE-based and ABR-based screening and are represented once in both the OAE and ABR columns; both patients passed the OAE test and failed the ABR test

NBHS relies on either OAE or ABR testing with pass/fail criteria. In our dataset, NBHS results were available for 73 patients. Of the 35 NBHS that utilized ABRs, 32 (91%) were failed and three (8.6%) were passed (Fig. 8). In contrast, only one of 24 (4.2%) OAE-based NBHS were failed while 23 of 24 (96%) were passed. In 16 cases, the NBHS method was not reported, and of those, 15 (94%) failed and one (6.3%) passed. Two patients are represented twice in Fig. 8 as they received both OAE-based and ABR-based NBHS, and both passed the former and failed the latter.

Thirty-two cases from 12 publications reported, typically in subjective terms, the efficacy of hearing aids (Table 3). None of the patients who used hearing aids received clinically meaningful benefit that improved conversational speech perception. Additionally, 17 publications provided information on the efficacy of cochlear implants in a total of 100 patients. Although the potential for quantitative analyses was limited due to the heterogeneity of the reported data, the outcomes suggest that cochlear implants reliably improved speech perception (Table 4).

**Table 3:** Hearing aid outcomes

Publication	Cases	Outcomes
Chiu et al. (2010)	6	“All the 6 homozygotes had limited benefits from hearing aids”
Costa et al. (2012)	2	“Could not tell whether the hearing aid was working or not” “The child obtained no benefits from the device”
Loundon et al. (2005)	1	“The mean threshold in free field… was 75 dB with hearing aids”
Marlin et al. (2010)	3	“Pure-tone hearing levels improved by 10… and 15 dB… with hearing aid amplification, but did not dramatically improve vocal audiometry performance”
Rouillon et al. (2006)	2	“No improvement was seen with the bilateral powerful hearing aid”
Runge et al. (2013)	2	“Aided benefit was reportedly limited to inconsistent increase in sound awareness”
Santarelli et al. (2015)	8	“Aided thresholds showed a marked improvement in pure tone sensitivity… however, they proved to be above the intensity range of conversational speech”
Santarelli et al. (2021)	2	“Both adult subjects had tried hearing aids without benefit”
Tang et al. (2017)	1	“He had worn hearing aids in both ears since… birth, but his ability to communicate was not improved”
Varga et al. (2003)	3	“All the children reported receiving no benefit from hearing aids”
Zadro et al. (2010)	1	“The child showed poor perceptive skills, only detection of sounds and words using hearing aids”
Zhang et al. (2013)	1	“6-month trial of conventional amplification failed to show benefit”

The outcomes of hearing aids reported in 32 individuals across 12 publications are summarized here. Reports were typically subjective, and no individual received from hearing aids benefit that improved conversational speech perception

**Table 4:** Cochlear implant outcomes

Publication	Cases	Outcomes
Gallo-Terán et al. (2006)	1	Appropriate language development at 26 months of age; does not require lip-reading
Iwasa et al. (2022)	36	≥ 2 years post-CI, for 6% CAP = 7, 79% CAP = 6, 9% CAP = 5, 3% CAP = 4, 3% CAP = 3; 47% had 20–29 dB hearing threshold, 42% had 30–39 dB threshold, 11% had 40–49 dB threshold
Kim et al. (2018)	10	Mean pre-CI CAP = 0.2; 3 months post-CI CAP = 2.2 (n = 10); 12 months post-CI CAP = 4.6 (n = 7); 24 months post-CI CAP = 5.4 (n = 5); 36 months post-CI CAP = 7 (n = 2)
Lee et al. (2022)	10	At mean of 24.1 months post-CI, mean CAP = 6.2
Loundon et al. (2005)	1	Pre-CI 75 dB hearing threshold, 4/40 MAIS; 12 months post-CI, 45 dB hearing threshold, 31/40 MAIS
Rodríguez-Ballesteros et al. (2003)	7	All “considered successful in terms of sound detection and communication skills”
Runge et al. (2013)	2	3 years post-CI on LNT Easy spoken word score, one patient had 80%, one patient had 44%
Rouillon et al. (2006)	2	One patient 18 months post-CI had 45 dB hearing threshold and 31/40 MAIS; one patient 3 years post-CI had 37 dB hearing threshold and 40/40 MAIS
Santarelli et al. (2015)	6	1–1.5 years post-CI, mean hearing threshold was 25–30 dB with 90–100% disyllable recognition
Santarelli et al. (2021)	5	Authors report improvements in speech perception for all 5 patients
Tang et al. (2017)	2	Both patients report improvement in speech perception and pronunciation
Varga et al. (2006)	2	Both patients report benefit
Wu et al. (2018)	10	Pre-op, median CAP = 1.5; 1 year post-CI CAP = 5; 5 year post-CI CAP = 6
Wu et al. (2020)	1	Authors describe outcome as successful; 3 years post-CI, enrolled in regular school
Zadro et al. (2010)	3	Authors report improvement for all patients
Zhang et al. (2013)	1	3 years post-CI, 25 dB hearing thresholds with excellent speech and language recognition, and is enrolled in regular school
Zhang et al. (2016a)	1	Temperature-sensitive case; 2 years post-CI, 93% open-set word recognition and 98% open-set sentence recognition

The outcomes of cochlear implants reported in 100 individuals across 17 publications are summarized here. One publication, Lee et al. (2022), did not contain patient-specific data. Nearly all reported cochlear implantations were considered successful

*CI* cochlear implant, *CAP* Categories of Auditory Performance, *MAIS* Meaningful Auditory Integration Scale, *LNT* Lexical Neighborhood Test

## Discussion

Two recent publications have made significant contributions to our understanding of otoferlin-related SNHL. Vona et al. (2020) reviewed both transgenic mouse models and human clinical literature to describe in detail the function and expression patterns of otoferlin isoforms, the spectrum of *OTOF* sequence variants and their impact on otoferlin function and phenotypes, and the clinical implications of this knowledge. Thorpe et al. (2022) combined audiometric data from original cases and cases reported in the literature to analyze the correlation between genotypic and phenotypic severity in 233 patients with biallelic, pathogenic *OTOF* variants. Here, we analyzed patient-specific data from 422 unique cases to characterize the genotype–phenotype relationship at the level of individual variants, the relationship between OAEs and multiple case characteristics, and the clinical outcomes of NBHS, hearing aids, and cochlear implants.

### Genotype–phenotype relationships

Our comprehensive literature review indicates that 85–90% of cases with biallelic, pathogenic or likely pathogenic *OTOF* variants are “typical” in that they display prelingual, severe-to-profound SNHL (Fig. 2). However, 10–15% of cases are “atypical” with mild-to-moderate, temperature-sensitive, unstable, or progressive phenotypes. These proportions deviate from those reported in studies of cohorts from geographically or ethnically homogeneous populations. Such studies typically describe relatively homogeneous *OTOF* genotypes and phenotypes likely due to evolutionary founder effects, though the nature of this homogeny differs between studies. For example, Rodríguez-Ballesteros and colleagues published two reports with a total of 57 cases, all of which had profound SNHL (Rodríguez-Ballesteros et al. 2003, 2008). The majority of these cases were of Spanish descent, 30 (53%) were homozygous for p.Q829X, and 19 (33%) were compound heterozygous for p.Q829X and another pathogenic variant. Two publications by Iwasa and colleagues described slightly more heterogeneous cases in Japan. The first contained 32 cases with phenotypic data, of which 31 (97%) had severe or profound hearing loss and 27 (84%) had at least one p.R1172Q allele (Iwasa et al. 2019). The second publication contained 63 cases with phenotypic data, of which 58 (92%) had severe or profound hearing loss and 58 (92%) had at least one p.R1939Q/W allele (Iwasa et al. 2022). In contrast to the Spanish and Japanese studies, a report of 22 Taiwanese cases described two thirds of their cohort as having moderate SNHL and only one third as having severe-to-profound SNHL (Wu et al. 2019). Twenty (91%) of these patients had at least one p.E1700Q allele, and of the 12 patients with longitudinal data, eight (67%) displayed progressive or fluctuant phenotypes. None of the cases from Wu et al. (2019) were included in our dataset because the publication did not contain any case-specific data. However, of the 29 p.E1700Q alleles in our dataset, 12 (41%) were associated with progressive phenotypes and four (14%) with temperature-sensitive phenotypes (Fig. 5C). Other studies have documented divergent rates of *OTOF* sequence variations or founder variants in individuals of Pakistani (Choi et al. 2009), Chinese (Zhang et al. 2016a), Turkish (Duman et al. 2011), French (Baux et al. 2017), and Ashkenazi Jewish descent (Fedick et al. 2016). In conjunction with our analyses, and in concurrence with Vona et al. (2020) and Thorpe et al. (2022), these studies demonstrate that *OTOF*-related phenotypes depend largely on genotypes, and that the prevalence of common genotypes can depend on the ethnicity or geography of a given population (Table 2).

Typical cases likely arise from two non-functional *OTOF* alleles, which produce proteins that are either truncated and rapidly degraded or carry variations that impair the protein’s ability to bind calcium or phospholipid membranes (Thorpe et al. 2022). Conversely, atypical cases likely occur when at least one allele produces a partially functional protein, which might be sufficient for facilitating calcium-dependent exocytosis but insufficient for replenishing and trafficking vesicles, resulting in rapid vesicle depletion and synaptic fatigue (Pangršič et al. 2010, 2012; Wynne et al. 2013; Santarelli et al. 2015; Vogl et al. 2015; Strenzke et al. 2016; Liu et al. 2023). This may explain why atypical phenotypes almost exclusively arise in cases with at least one NT allele—in our dataset, 22% of NT/NT cases and 14% of NT/T cases were phenotypically atypical (Fig. 4A, B), while just 2.7% of T/T cases were atypical (Fig. 4C). These proportions are remarkably similar to the 22% of missense/missense, 14% of missense/loss-of-function, and 2.3% of loss-of-function/loss-of-function cases Thorpe et al. (2022) described as “less than severe,” though both their analysis and ours drew data from many of the same publications.

The locations of NT sequence variations and the frequencies with which they are reported and associated with particular phenotypes may offer insight into the functional importance of specific protein domains (Fig. 5). Presynaptic calcium plays a central role in exocytosis by regulating vesicle priming, fusion, and replenishment via interactions with multiple proteins, including otoferlin (Beutner et al. 2001; Vona et al. 2020). In our dataset, nearly 50% of reported NT alleles occurred in C_2_ domains, suggesting the importance of calcium-binding in otoferlin function (Pangršič et al. 2012; Fig. 5B). However, the functionality of each otoferlin C_2_ domain is debated. C_2_A does not bind calcium, but it facilitates endocytic membrane retrieval and binding with synaptic proteins (Helfmann et al. 2011; Liu et al. 2023). Liu et al. (2023) recently identified a short otoferlin isoform lacking C_2_A in mouse IHCs. They demonstrated that transgenic mice expressing only the short isoform and not the canonical isoform had intact hearing thresholds despite abnormal ABRs and reduced sustained exocytosis at the ribbon synapse. If expression of this short isoform is confirmed in humans, it may help explain why we identified no pathogenic NT variants in C_2_A (Fig. 5B; Liu et al. 2023). The calcium-binding abilities of C_2_B and C_2_C, which contain five (1.2%) and 24 (5.9%) NT alleles, respectively, in our dataset, are debated (Johnson and Chapman 2010; Pangršič et al. 2012; Padmanarayana et al. 2014; Meese et al. 2017; Vona et al. 2020). However, there is consensus that C_2_D, C_2_E, and C_2_F, which contain 26 (6.3%), 27 (6.6%), and 74 (18%) NT alleles, respectively, in our dataset, bind calcium and are functionally important (Vona et al. 2020). Sequence variants in C_2_E, including p.I1573T, are notable for being associated predominately with atypical phenotypes (n = 17 of 27 C_2_E alleles, or 63%; Fig. 5B, C). Interestingly, the putative C_2_ domain, C_2_de, was the third-most common location for NT variants to be reported with 45 (11%) alleles in our dataset, lending support for its hypothesized functionality (Pangršič et al. 2012; Vona et al. 2020; Fig. 5B). Additionally, the number of times NT variants occurring between C_2_F and the transmembrane domain were reported (n = 143), and the proportion of those alleles associated with typical severe-to-profound phenotypes (n = 135; 94%), suggest that conservation of this region is critical for otoferlin functionality (Fig. 5B).

In addition to the ten alleles associated with temperature-sensitive phenotypes described in the results section, one additional variant, p.I515T, was identified in two siblings with temperature-sensitive DFNB9 phenotypes (Varga et al. 2006). However, these cases were not included in our analyses because biallelic sequence variations could not be identified. In other reports, p.I515T has been associated with a typical phenotype (Leal et al. 1998; Mirghomizadeh et al. 2002). Varga et al. (2006) also reported identifying the p.R1157Q variant in their cohort, though they classified it as a benign polymorphism and did not specify in which subject(s) it was found. Later, Marlin et al. (2010) identified three siblings with temperature-sensitive phenotypes homozygous for both p.R1157Q and p.E1804del. The p.R1157Q variant has been identified in one other patient, though its pathogenicity remains uncertain (Dallol et al. 2016). Consequently, the link between temperature-sensitive phenotypes and the p.I515T, p.R1157Q, and p.E1804del variants is not completely clear. However, the association between temperature-sensitive phenotypes and the eight other variants (p.G541S, p.G614E, p.R698T, p.R1080P, p.R1607W, p.P1628T, p.E1661K, p.E1700Q) described in the results is more certain.

### Otoacoustic emissions

Over 80% of cases with OAE data had present, reduced, or present-then-absent OAEs (Fig. 6A), suggesting that a portion of OHCs are functional in a majority of patients with otoferlin-related SNHL, at least in early childhood. When stratified by age, our dataset suggests that OAEs, and thus OHC functionality, are less common in older individuals and may be lost with age. OAEs were absent in only 13% (n = 17 of 136) of children 10 years of age or younger, but they were absent in 40% (n = 16 of 40) of individuals over 10 (Fig. 6B). Although the proportion of cases with OAEs was fairly constant among age bins under 10 years in our dataset, two longitudinal studies have reported OAE deterioration in young children. Kitao et al. (2019) described 10 patients (not included in our dataset due to a lack of case-specific data) with biallelic *OTOF* variants and showed that OAEs rapidly deteriorated in all patients during the first few years of life. Similarly, Thorpe et al. (2022) conducted serial OAE testing in four patients between one and five years of age and found that OAEs deteriorated in five of eight ears during that time.

OAEs were more likely to be absent in cases with typical phenotypes (n = 35 of 154, or 23%) than in cases with atypical phenotypes (n = 4 of 39, or 10%), and none of the mild (n = 10) or temperature-sensitive (n = 13) cases had absent OAEs (Fig. 6C). Additionally, T/T cases were more than 2.5 times more likely to have absent OAEs than NT/NT or NT/T cases (Fig. 6D). Our analyses stratifying OAE data by age and either phenotype (Fig. 7A–D) or genotype (Fig. 7E–G) must be interpreted cautiously due to small sample sizes in many categories and methodological variability in how sample populations were selected between publications. Nevertheless, the data suggest that NT/NT cases with OAEs tend to be older (median age five years) than NT/T or T/T cases with OAEs (median age three years for both), that T/T cases with OAEs tend to be younger (median age three years) than those without OAEs (median age 13 years), and that profound cases with OAEs tend to be younger (median age four years) than those without OAEs (median age seven years). All these inferences support the hypotheses that OAEs may be lost over time, and that OAEs are more likely to be lost in cases with more severe genotypes and phenotypes. However, a wide range of ages, often spanning from less than one to more than forty years old, were represented among patients both with and without OAEs in most genotype and phenotype categories. This suggests that other variables, including environmental factors like noise exposure, may contribute to OAE loss in otoferlin deficiency. Elucidating the precise mechanisms driving OAE loss is a crucial objective for future research. Prospective studies with serial OAE testing would be particularly informative.

### Clinical implications

Given the genotype–phenotype associations described above, an individual’s specific genotype can inform clinical guidance. Even if a patient’s sequence variations are novel, some insight may be gleaned from both the severity (NT vs. T) of the variant and the region of the protein it affects. For instance, a child born with mild SNHL and NT variants in the C_2_E domain might have a stable phenotype, whereas mild SNHL in a child with NT variants between C_2_E and C_2_F may be more likely to progress (Fig. 5B).

It is also important to recognize that different clinical techniques can yield divergent results. For instance, 30 of 154 (19%) cases with absent ABR at maximum stimulation displayed atypical phenotypes as measured by audiometric hearing thresholds, including seven (4.5%) with mild, eight (5.2%) with moderate, and eight (5.2%) with temperature-sensitive hearing loss (Fig. 2E). This discrepancy between ABR and tone detection thresholds likely occurs because *OTOF* variants can disrupt temporal dynamics of vesicular exocytosis necessary for synchronous postsynaptic neural activity (measured by ABR) while still allowing enough dysregulated exocytosis for sound detection (measured by audiometric hearing thresholds; Pangršič et al. 2010; Santarelli et al. 2015, 2021; Strenzke et al. 2016; Vona et al. 2020; Liu et al. 2023). Neural synchrony is necessary for encoding auditory signals with high temporal fidelity. Consequently, patients with mild or moderate tone detection thresholds and absent ABRs may have more severe functional deficits (e.g., speech perception) than suggested by behavioral audiometry (Santarelli et al. 2013).

Similarly, NBHS relies on either OAE testing or more time-intensive ABR testing. Unfortunately, the majority of babies born with *OTOF*-related SNHL or other forms of ANSD pass OAE-based NBHS, leading to delayed diagnoses and interventions (Fig. 8; Kim et al. 2018; Iwasa et al. 2022). To reliably detect ANSD, NBHS must include ABR testing (Fig. 8; Rouillon et al. 2006; Vona et al. 2020). Early detection of hearing loss enables early intervention (e.g., before two years of age) with medical devices during critical periods of brain plasticity and language acquisition, which improves outcomes (e.g., language skills development) for children with congenital SNHL (Nicholas and Geers 2007; Ching et al. 2017; Kral et al. 2019; Azaiez et al. 1993). Consequently, leaders in the field have called for inclusion of ABR in newborn screening and early genetic testing of all children with SNHL (Shearer and Smith 2015; Shearer et al. 2019; Thorpe and Smith 2020; Vona et al. 2020).

Hearing aids are the first-line intervention for children diagnosed with *OTOF*-related SNHL (Azaiez et al. 1993). However, the 32 cases with hearing aid outcomes reported in the literature suggest that patients with otoferlin-related SNHL do not receive meaningful benefit from so-called “hearing aid trials,” which are currently required for cochlear implant eligibility in all types of congenital SNHL, regardless of etiologic diagnosis (Table 3). In one report, although eight patients had improvements in pure-tone hearing thresholds, their aided thresholds remained “above the intensity range of conversational speech” and all eight went on to receive cochlear implants (Santarelli et al. 2015). Notably, it has been hypothesized that *OTOF* variants might render OHCs more susceptible to noise-induced damage, and that hearing aids may thereby accelerate the loss of OAEs through constant exposure to high intensity stimuli in an inner ear with normal cochlear amplification mechanisms (Rouillon et al. 2006; Kitao et al. 2019; Vona et al. 2020).

Unlike hearing aids, cochlear implants provided speech perception benefit in nearly all 100 cases reported in our dataset (Table 4). This is consistent with the conclusions of a recent review of cochlear implant outcomes in patients with biallelic *OTOF* variants (Zheng and Liu 2020). Given the reliable benefit of cochlear implants in these patients, early genetic screening may facilitate prompt cochlear implantation and better long-term outcomes (Shearer and Hansen 2019; Azaiez et al. 1993; Carlson et al. 2023). Furthermore, the stark contrast between hearing aid and cochlear implant outcomes warrants further investigation with prospective studies and a re-evaluation of the existing evidence to support hearing aid trials in children with genetically confirmed otoferlin deficiency.

Due to intact cochlear and neural anatomy in most patients with otoferlin-related SNHL, experimental gene therapies that restore functional otoferlin to IHCs might provide an opportunity for instating the use of natural cochlear processes, physiologic hearing, and potentially superior outcomes compared to management with medical devices (Vona et al. 2020). This approach has proved promising in preclinical studies utilizing animal models of otoferlin deficiency (e.g., otoferlin-knockout mice; Akil et al. 2019; Al-Moyed et al. 2019). Like interventions with medical devices, future gene therapy interventions are also likely to be most effective if initiated at an early age, but not only to coincide with critical periods of brain plasticity and language development. The progressive deterioration of OHC responses (i.e., OAEs) is a key feature of the natural history of otoferlin deficiency (Kitao et al. 2019; Thorpe et al. 2022), and it remains unknown the degree to which OHC and OAE preservation will affect the efficacy of gene therapy. Similarly, it is unclear whether cochlear damage from the surgical implantation of electrodes will preclude the use of gene therapy in ears that previously underwent cochlear implantation (Carlson et al. 2011; Bas et al. 2012). These questions will be important to investigate in the event that gene therapies are shown to be effective treatments for congenital SNHL.

### Limitations and recommendations for future research

Our analyses, and the literature on which they are based, have several important limitations, which raise considerations for future research. The majority of the literature on otoferlin-related SNHL is comprised of case reports or cross-sectional studies with small sample sizes limited by the frequency of *OTOF* variants in a given population. Although challenging to conduct, additional prospective studies with longitudinal data would be valuable for further elucidating the natural history of this disorder. Additionally, most studies were conducted in specific geographic regions (e.g., Japan, Spain), which may differ in clinical practices and *OTOF* allele frequencies. Consequently, foremost among the potential confounds of our analyses is the disproportionate influence of a small number of papers with large sample sizes derived from populations that may not be representative of the global population. For example, 55 of the 422 cases in our dataset came from 13 consanguineous Pakistani families described in Choi et al. (2009). Additionally, cases with atypical phenotypes—particularly temperature-sensitive and progressive cases—may be more likely to be reported in the literature than cases with typical phenotypes due to their novelty, which could result in these phenotypes being overrepresented in our dataset.

Our analyses were complicated by a large amount of methodological variability between studies and, in some instances, unclear descriptions of methodological details. Studies differed in their clinical techniques (e.g., free field vs. ear-specific air and bone conduction), phenotypic bins (e.g., hearing threshold cutoffs), diagnostic criteria (e.g., definitions of present vs. reduced OAEs), inclusion/exclusion criteria, and genetic sequencing methodologies. For instance, some cases were described as having hearing thresholds “greater than” a certain value (e.g., ≥ 80 dB); as we used the lower limit (e.g., 80 dB) to assign a phenotype to these cases, our analysis may overestimate the number of severe cases and underestimate the number of profound cases. Methodological variability is inevitable, but detailed reporting of methodological techniques and case-specific data (rather than group data only) ensures transparency, facilitates accurate interpretation, and enables subsequent quantitative reviews and meta-analyses.

Moving forward, one open question is how, if at all, *OTOF* sequence variants affect the vestibular system. Otoferlin is expressed in vestibular hair cells and there are limited reports of patients with both *OTOF* variants and vestibular dysfunction, but no causal relationship has been established (Varga 2003; Varga et al. 2006; Iwasa et al. 2019; Gao et al. 2021). It is also notable that we documented 42 patients heterozygous for a single *OTOF* variant and with phenotypes compatible with otoferlin-related SNHL (Rodríguez-Ballesteros et al. 2003; Varga et al. 2003, 2006; Romanos et al. 2009; Chiu et al. 2010; Wang et al. 2010, 2011; Matsunaga et al. 2012; Iwasa et al. 2013), in addition to 14 heterozygous patients with late-onset, unilateral hearing loss that the authors suspected may have been related to otoferlin deficiency (Gao et al. 2021). This observation raises the possibility that some molecular detection methods, such as those commonly employed in older publications, may not reliably detect sequence variants in *OTOF*. Therefore, it may be worth re-sequencing these ostensibly heterozygous patients using more sensitive next-generation sequencing techniques. Alternatively, there may be more unidentified exons (Ranum et al. 2019) or isoforms (Liu et al. 2023), or heterozygosity might cause a subclinical reduction in otoferlin levels (Pangršič et al. 2010) that leaves individuals more susceptible to other environmental or genetic insults. Additionally, the complexity of otoferlin and the rarity of cases with biallelic *OTOF* variants make it difficult to discern the pathogenicity of certain variants, particularly those with only one documented occurrence. Given the rarity, genetic heterogeneity, and phenotypic spectrum of otoferlin-related SNHL, every case described in the literature and each variant (regardless of pathogenicity) reported to online databases is valuable. Through the collective effort of researchers and clinicians around the world, *OTOF*-related hearing loss will continue to be better understood and more effectively treated in the future.

## Data Availability

All data relevant to the results and conclusions of this study were derived from published literature as referenced within the article or described in the methods.
